# A Sound Prediction: EEG-Based Neural Synchrony Predicts Online Music Streams

**DOI:** 10.3389/fpsyg.2021.672980

**Published:** 2021-07-27

**Authors:** Nikki Leeuwis, Daniela Pistone, Niels Flick, Tom van Bommel

**Affiliations:** ^1^Unravel Research, Utrecht, Netherlands; ^2^Tilburg University, Tilburg, Netherlands; ^3^Applied Cognitive Psychology, Utrecht University, Utrecht, Netherlands

**Keywords:** electroencephalogram, neuromarketing, neuroforecasting, music, neural synchrony, popularity prediction, inter-subject correlation, EEG

## Abstract

Neuroforecasting predicts population-wide choices based on neural data of individuals and can be used, for example, in neuromarketing to estimate campaign successes. To deliver true value, the brain activity metrics should deliver predictive value above and beyond traditional stated preferences. Evidence from movie trailer research has proposed neural synchrony, which compares the similarity of brain responses across participants and has shown to be a promising tool in neuroforecasting for movie popularity. The music industry might also benefit from these increasingly accurate success predictors, but only one study has been forecasting music popularity, using functional magnetic resonance imaging measures. Current research validates the strength of neural synchrony as a predictive measure for popularity of music, making use of electroencephalogram to capture moment-to-moment neural similarity between respondents while they listen to music. Neural synchrony is demonstrated to be a significant predictor for public appreciation on Spotify 3 weeks and 10 months after the release of the albums, especially when combined with the release of a single. On an individual level, other brain measures were shown to relate to individual subjective likeability ratings, including Frontal Alpha Asymmetry and engagement when combined with the factors artist and single release. Our results show the predictive value of brain activity measures outperforms stated preferences. Especially, neural synchrony carries high predictive value for the popularity on Spotify, providing the music industry with an essential asset for efficient decision making and investments, in addition to other practical implications that include neuromarketing and advertising industries.

## Introduction

The application of neuroscience methods to marketing could provide marketeers with new information that is not accessible by conventional marketing research methods (Ariely and Berns, 2010). This line of thought is also called neuromarketing or consumer neuroscience and has expanded tremendously since its discovery (Plassmann et al., 2012; Alvino et al., 2020). Consumers are often unwilling or unable to correctly express their preferences explicitly. Their stated preferences are biased by the conscious cognitive control over the underlying subconscious response, and consequently, the measurement of explicit choices will not reflect their true preferences (Christoforou et al., 2017).

The lack of introspective capabilities of consumers into their own true preferences is evident when consumers are asked to explicitly reflect on their buying process. For example, the act of reflection actually modified their perception of the choice also called the mere-measurement effect (Morwitz and Fitzsimons, 2004). Other biases that obscure sound decision making include social conformity (Zaki et al., 2011), which causes high variability in subjective ratings. Moreover, Dijksterhuis (2004) showed that distracting consumers, thereby forcing them to make a decision without conscious awareness, resulted in better decisions compared to consciously choosing consumers. To overcome these biases, brain research has been found a fruitful avenue for revealing true preferences.

The line of neuroscientific research where group level interest is predicted from brain activity is called neuroforecasting by Knutson and Genevsky (2018). Their review examines the ability of predicting population-wide choices based on the information of the individual, assuming some choice components may generalize across individuals, and others may not. This paradigm is based on observations that neural representations are – to some extent – modality-general and individual-invariant (Chan et al., 2020) and can therefore be predicted from individual neural responses. Indeed, forecasting the behavior of a large group has been shown to outperform individual stated preferences, values and choices, including viral marketing success (Motoki et al., 2020), the decision to keep watching online videos (Tong et al., 2020), and the success of anti-smoking ads and campaigns (Falk et al., 2012; Schmälzle et al., 2020).

Besides these applications, an impressive body of research is devoted to predicting the success of movies with neuroscience methods (e.g., Hasson et al., 2008b; Dmochowski et al., 2012; Barnett and Cerf, 2017). For example, Boksem and Smidts (2015) found that higher box-office revenue sales of a movie could be forecasted from increased activity at gamma frequencies at fronto-central sites during the viewing of a movie trailer and that the likeability scores on an individual level could be related to an increased electroencephalogram (EEG) activity in the beta range on mid-frontal sites. This demonstrated that even for personal preference, EEG data adds predictive value beyond the respondents’ own ratings, stressing the fact that implicit measurements contain unique information that is not accessible with traditional research methods (e.g., self-reports). Their results highlight the added value of EEG on a population-level prediction; box-office revenue predictions improved significantly when the model included EEG data instead of only subjective data.

And this is not the only study where brain activity during exposure to a movie trailer is shown to remarkably increase the accuracy of forecasts of individual and population-wide popularity. Hasson et al. (2008b) proposed the term neurocinematics in order to indicate that the neuroscientific quantitative measurement of viewers’ engagement might impact the style of filmmakers and enable the film industry to better assess its products. Now, a decade after the term has been introduced, neurocinematics has matured in the light of numerous studies demonstrating the implicit experience of the audience outperforms traditional methods in revenue prediction.

More interestingly, when we turn to the neural indicators underlying the prediction of market level outcomes, several studies have indicated a comparable measure of brain activity. It has been found that the similarity between viewers’ brain activity during the viewing of a movie (trailer) was predictive of levels of attention (Hasson et al., 2008b), emotional arousal (Nummenmaa et al., 2012), engagement (Dmochowski et al., 2012; Chan et al., 2019), social buzz (Dmochowski et al., 2014), future recall (Barnett and Cerf, 2017), memory encoding (Hasson et al., 2008a), personal liking (Chan et al., 2019), or box-office revenues (Barnett and Cerf, 2017; Christoforou et al., 2017). Hasson et al. (2004) were the first to compare the brain responses of one respondent to that of others. By comparing the brain regions across all pairs of respondents, they could identify similar brain responses across the subject pool while they were watching *The Good, the Bad, and the Ugly*. Besides similarity in visual and auditory cortices, frontal and parietal regions also showed similar responses, indicating that the movie also engaged neural patterns associated with narrative and emotional processing.

Where Hasson et al. (2004) referred to the comparison over brain activity over multiple subjects as synchronization, other studies use the terms of alignment (Golland et al., 2017), inter-subject correlation (ISC; Nummenmaa et al., 2012) or between-subject correlation (Haxby et al., 2011), cross-brain correlation (Barnett and Cerf, 2017), neural reliability (Dmochowski et al., 2014), or consistency (Lankinen et al., 2014). In the present research, these terms will be used interchangeably although neural synchrony is preferred.

Schmälzle and Grall (2020) provided an extensive review of research on the similarity of neural responses. Methods that have been employed include magnetoencephalography (MEG; Lankinen et al., 2014) and electroencephalography (EEG; Barnett and Cerf, 2017) but most studies focused on functional magnetic resonance imaging (fMRI; e.g., Hasson et al., 2008b; Dmochowski et al., 2014; Chan et al., 2019). These brain-to-brain similarities can be understood as commonalities between the signal processing of the observers (Schmälzle and Grall, 2020), a collective engagement. This could be explained by the idea that efficient communication requires a successful translation from one individual to another (Hasson et al., 2004), and therefore, similar brain activity should indicate similar experience, needed for efficient communication purposes (Stephens et al., 2010). This is highlighted by studies that deliberately messed up the communication, either by showing unstructured video footage (Dmochowski et al., 2012) or providing manipulated background information (Yeshurun et al., 2017), where indeed neural synchrony was decreased.

Whereas the prediction of movies has gained sufficient research interest, popularity of music has been sparsely evaluated. As it is essential to anticipate the behavior of large audiences to movies for the film industry, so might the music industry profit from forecasting the sales and digital streams of their albums and singles. The rationale for prediction of movie popularity is provided by the urge to detangle the complex interdependencies of the creative efforts, cast and promotional budgets, as they might all significantly impact the chances of success for a certain movie (Elberse, 2007). However, these arguments do not only hold true for movie trailers; they do also apply to the music industry.

The research into predicting music popularity from brain activity started with a study of Berns et al. (2010) that investigated the effect of social influence on music preference with fMRI. The researchers brought 32 subjects to the laboratory to listen to relatively unknown music that they found on MySpace. The popularity of these songs was established by the number of downloads from MySpace. Two years later, Berns and Moore (2012) heard some of these initial unknown songs suddenly being played on the radio, which led them to reexamine their dataset to investigate whether they could have foreseen which of these songs would become popular. Although they found that the subjective ratings of respondents were not related to the real sales data, they could positively correlate brain activity measured with fMRI within the ventral striatum to the number of units sold. This additional analysis subsequently validated that music popularity can be predicted from brain activity. The line of research into music popularity, however, seems to have ended there.

There have been some studies employing neural synchrony measures on musical experience, e.g., Trost et al. (2015) and Sachs et al. (2020) both studied how the affective processing in fMRI during listening to music is related to subjective ratings of valence, showing that the degree of neural synchrony in brain regions was driven by separate features of music; e.g., synchronized amygdala responses derived from features, such as dissonance, while similar insula responses were derived from acoustic density (Trost et al., 2015). The subjective experience of music was found to relate to neural synchrony in the affective processing circuit (Sachs et al., 2020), and Kaneshiro et al. (2020) showed that basic musical features, such as rhythm and melody, elicited significantly higher neural correlation compared to scrambled musical samples. Additionally, Czepiel et al. (2020) calculated the ISC during three live concerts and showed that tempo was consistently related to increased neural synchrony as well as phrase repetitions, transitional passages, and boundaries.

The application of neural synchronization to music actually makes sense as it is shown that brain waves synchronize with visual and auditory frequencies (e.g., Tyler et al., 1978; Toiviainen et al., 2010). There have been multiple studies investigating neural similarity reactions to music in both fMRI and EEG and even one study that related neural synchrony between the musician and the listener to subjective likeability of the violin concert (Hou et al., 2020). However, besides Berns and Moore (2012) none of them have tied these measures to the general popularity of music.

As is the case with movies, everyone has musical preferences and it provides a perfect integration of narrative and emotion – the aspects that are assessed with neural similarity. In addition, Vogel (2020) estimated that perhaps only 10% of new releases end up making profit for a record label, compensating the financial loss that happens typically on 85% of releases. Labels will encourage excessive production of material without prior knowledge on what will succeed, essentially just diversifying their bets. Digital streaming now accounts for 85% of music industry revenue in the United States (Recording Industry Association of America, 2020). Standing out within this digital music marketplace may be harder compared to traditional CD selling (Simon, 2019; Kaimann et al., 2021). This might urge the need to focus on efficient distribution of promotional budgets even more.

Combining this urge to distribute marketing budgets efficiently with the neural similarity neuroforecasting metric above and the promising results that followed from movie trailer analysis, we propose the use of neural synchrony as a measure of music popularity, making use of EEG to capture moment-to-moment neural similarity between respondents while they listen to music.

Since the study of Berns and Moore (2012) was conducted with fMRI, the validation of EEG in predicting music popularity has yet to be established. The uncomfortable, noisy environment of fMRI measures might have a substantial negative impact on the external validity of choices and the cognition of the respondent, whereas an EEG is wireless, lightweight, and thereby less invasive, additionally less expensive than fMRI and therefore often used in commercial neuromarketing settings. Due to the mobile headset and non-claustrophobic environment, the EEG measurements allow for less obtrusive measurement, and therefore less supposed influence on behavior than fMRI. However, an essential trade-off needs to be made between spatial and temporal resolution. As Dmochowski et al. (2012) state: The fMRI shows if neural activity significantly correlates in response to a common stimulus, but it is unable to show *precisely when* this synchronization occurs. This temporal aspect of EEG is essential as the predictive information in this analysis is carried by the simultaneous timing of responses.

Therefore, the current study aims to further validate the strength of neural synchrony as a predictive measure for popularity of music, which strongly follows from the previous literature. This research fills the void between the fMRI-based music prediction of Berns and Moore (2012) on the one hand and EEG-based neural similarity studies on movie trailers (e.g., Hasson et al., 2008b; Dmochowski et al., 2012; Barnett and Cerf, 2017) on the other hand. Meanwhile, this research shows a new application and validates this approach for a whole new branch that will benefit extensively from successful neuroforecasting. The predictive value of neural synchrony is compared to the sample’s stated preferences, and additionally, the neural measures related to these personal likings are also evaluated. Since neural synchrony is frequently interpreted as a measure of emotional engagement of users (e.g., Dmochowski et al., 2012; Chan et al., 2019), an additional intra-subject measure of engagement was included in the study. On the group level, this relationship was hypothesized to be validated, though on an individual level, we expected engagement might be related to likeability since emotional engagement relates to memory encoding and consequently personal purchase decisions (Sebastian, 2014). Another intra-subject metric was proposed from the neuromarketing literature, namely Frontal Alpha Asymmetry (FAA), which is related to stated pleasantness (Davidson et al., 1990; Vecchiato et al., 2011; Briesemeister et al., 2013) but also enables to predict larger audience popularity (Shestyuk et al., 2019), performing better than subjective ratings to predict sales (Baldo et al., 2015). It was therefore hypothesized that FAA would relate to subjective ratings, and additionally would generalize to the preferences of the larger audience.

Thus, in the present research, neuroforecasting will be evaluated on a group level using neural synchrony, FAA, and engagement. The predictive value will be established above and beyond stated preferences as it is important to compare predictive value to a baseline method. These stated preferences, on the other hand, will also be submitted to neuroforecasting on an individual basis; showing that both FAA and engagement are related to the personal subjective ratings.

## Materials and Methods

### Participants

Thirty-one people participated in the research. The participants were recruited from the Unravel Research participants database from a convenience sample and they received a monetary compensation for their participation. In total, 24 women and seven men between the ages of 19 and 65 (mean ± std. = 27.7 ± 11.6) participated. All the participants were right-handed, reported to have no psychological disorder and signed an informed consent prior to participation. One participant was removed from the data due to an error during data acquisition, which left the sample with 30 participants (mean ± std. = 26.87 ± 10.8).

### Stimuli

Fragments of the songs of two albums were used as stimuli during this study. The two albums were the R&B album called “It Was Good Until It Wasn’t” by Kehlani and the pop album called “How I’m Feeling Now” by Charli XCX. The albums were chosen because of their convenient release date and because they belong to different music genres. The R&B album contained 13 songs and the pop album contained 11 songs. The songs were sampled by the researcher into 24 s fragments and converted to MP3 format. The fragments were subjectively sampled and contained the most distinctive part of the song, usually the chorus and/or the hook. The stimuli were presented using the iMotions (2019) software.

### Experimental Procedure

The research started a few days after the albums’ release data to minimize participants’ familiarity of the songs. The participants were asked not to drink caffeine-containing beverages prior to the experiment.

At the onset of testing, the participants were seated in front of a computer screen and briefed on the objectives of the study. The participants were told that they would listen to and rate fragments of songs, while their brain activity is measured by an EEG device. Also, the participants were instructed to minimize their head movements in order to prevent EEG-data artifacts.

At the beginning of the experiment, participants were asked to rank the following music genres from 1 (“music genre you like the best”) to 6 (“music genre you like the least”): rock, pop, alternative, Hip Hop/rap, jazz/blues, and R&B.

Then, each participant was exposed to 24 song fragments. After each fragment, the participants were asked to rate the fragment based on how much they liked it. The ratings were done by using the 1–5-star scaling system. Each rating began with zero stars, and the participants could not continue without the rating, in order to prevent missing data. The order of the albums was counterbalanced, and the songs were played in a randomized order within the album to prevent order effects.

After listening to and rating all fragments of an album, the participants were asked if they have listened to the album prior to this research. This was done to ensure that the songs were new to every participant. When the experiment was completed, the participants were debriefed and thanked for their participation. The experiment was conducted following the principles outlined in the Declaration of Helsinki.

### EEG Acquisition

Brain activity was recorded using the wireless hardware system B-Alert X10 EEG including nine channels following the International 10/20 system of electrode placement (F3, Fz, F4, C3, Cz, C4, P3, POz, and P4). Two electrodes were placed as a reference on the mastoid bones behind both ears. Conductive gel was applied between the scalp and the electrodes, in order to keep the impedance below 40 kΩ. The EEG signals were recorded continuously throughout the experiment at 256 samples per second by the software iMotions (2019). After each participant, the channels were sanitized with alcohol.

### Metric Calculations

#### Preprocessing

Preprocessing of EEG data is performed within the decontamination algorithms of iMotions. Data was Notch filtered at 50 Hz and high-pass Butterworth filtered at 0.1 Hz. Artifacts were automatically removed when the signal amplitude exceeded 400 μV.

#### Power Spectral Density

The EEG signal is described by the distribution of power into frequency components. Power spectral density (PSD) is calculated by the Fast Fourier transform on all available electrodes. Welch window width was 0.5, and sliding window width is 500 ms, with step size 250 ms. The data was decomposed into frequency bands delta (1–3 Hz), theta (4–7 Hz), alpha (8–12 Hz), beta (13–25 Hz), and gamma (26–40 Hz) by averaging over the included frequencies. The R code to execute this was provided by the iMotions (2019).

#### Neural Synchrony

Neural synchrony is calculated with a custom R notebook (R Core Team, 2019). The calculation is derived from the previous studies on the inter-subject correlation (ISC) in either fMRI (Hasson et al., 2008a) and EEG studies (Dmochowski et al., 2014; Barnett and Cerf, 2017; Christoforou et al., 2017). Our approach most strongly resembles that of Barnett and Cerf (2017) where pairwise correlations are calculated for every electrode site and time step, which are then averaged to derive a single value of neural similarity. Alpha activity was evaluated in Barnett and Cerf (2017) as it is associated with attention to visual stimuli (Klimesch, 2012) and this frequency band was also employed here. Electrode selection followed the notion of Barnett and Cerf (2017) who showed that C4 and Cz were the most important electrode when predicting weekly ticket sales, thus we decided to select all central electrodes (C3, Cz, and C4).

The PSD over the central electrodes in the alpha band was correlated over time pairwisely for all possible pairs of participants. The steps window width is 1,000 ms, taking 500 ms steps to smoothen the line in qualitative analysis. This indicates that the underlying data results from −750 to +750 ms of the time point concerned. The pairwise correlations of all subjects are absolute and averaged over time. Then this average time series is averaged, resulting in one value per stimulus. The synchrony was multiplied by 100 for interpretability. Thus the equation follows where T is the number of time steps, i.e., the total duration of the musical sample divided by 250 ms, N is the number of subject, and $X_{i\left[t-2:t+2\right]}$defines the matrix of EEG activity of participant i in time *t* ± 500 ms over the three electrodes:

$$\frac{1}{T}\sum_{t=1}^{T}\frac{1}{N\left(N-1\right)}\sum_{i=1}^{N}\sum_{j=1,j\neq i}^{N}\left|cor\left(X_{i\left[t-2:t+2\right]},\;X_{j\left[t-2:t+2\right]}\right)\right|\;$$

#### Frontal Alpha Asymmetry

The PSD for alpha in channel F4 is log transformed, and the log transformed PSD in alpha frequency at channel F3 is subtracted. The R code to execute this was provided by iMotions and works following this equation:

$$\ln\left(PSD_{F4}\right)-\ln\left(PSD_{F3}\right)$$

For each subject, the values are averaged over time. Then for the group comparison, these are aggregated for every stimulus.

#### Engagement

Before starting the experiment, an alertness and memory profiler (AMP) test is conducted on each participant. This test is provided by ABM (Advanced Brain Monitoring, Inc., Carlsbad, CA). The test consists of three tasks: the three-choice vigilance task, visual psychomotor vigilance task, and auditory psychomotor vigilance task. Each task takes 3 min to complete.

Measures of cognitive states are provided by discriminant function analysis of the EEG signals, which is provided by ABM and runs within iMotions automatically. Based on the EEG recording during the AMP tests, the model provides the probabilities of cognitive states. This study included high engagement (Berka et al., 2007). These commercially available metrics have been shown to be valuable in both marketing and human performance areas (Bernhardt et al., 2019).

### Data Analysis

Spotify streams were divided by a million for interpretability. The data was scanned for outliers using their deviation from the mean and multidimensional outliers with a Mahalanobis distance. Outliers deviating more than three standard deviations from the mean were removed from the analysis.

Before correlational analyses were performed, variables were checked on normality using Shapiro-Wilk tests. When both variables were normally distributed, Pearson correlation was implemented. When one of them was not, Kendall correlation coefficients were calculated. Kendall was preferred over Spearman rank since it is considered to be more robust and efficient compared to Spearman correlation (Croux and Dehon, 2010).

#### Group Level Analysis

Firstly, a correlational analysis was performed in order to establish the relationship between the neurological measurements and Spotify streams. In addition, the relationship between subjective ratings and the number of streams was established, to serve as a baseline.

Multiple comparisons were corrected with Bonferroni correction. The analysis encompasses two dependent variables that are compared on subjective ratings and three neural measures. So in total, eight analyses were done, setting the significance level at 0.006.

General popularity was evaluated by linear regression on the number of streams in Spotify both 3 weeks and 10 months after album release. The assumptions underlying linear regression were tested with a Shapiro-Wilk test for normality of residuals, Breusch-Pagan test checking the assumption of homoscedasticity (constant variance; Zaman, 2000). Linearity assumptions were checked visually.

When the assumptions were not met, natural log transformation was done in order to correct the distribution of the dependent variables, following Dmochwoski et al. (2014) who did the same for heavy-tailed Twitter distributions and Berns and Moore (2012) with the skewed number of music downloads. However, this has consequences regarding the interpretation of the model coefficients (Changyong et al., 2014). Where one would expect that taking the exponent of the coefficient would explain the contribution of that variable, Changyong et al. (2014) argue to transform coefficients by exp. (b + σ^2^/2). To increase interpretability of the results, the log transformation was only applied when this was needed through violated assumptions.

#### Individual Level Analysis

Since the individual liking is indicated by scores on a five point Likert scale, non-parametric Kendall correlation coefficients were calculated between psychological parameters and likeability scores. Multiple comparisons were corrected with Bonferroni correction. The analysis encompasses subjective ratings and two neural measures. Thus, two correlational analyses were done, setting the significance level at 0.025.

The genre that participants indicated as their personal preference in the first question was divided into a binary state. As most participants in the sample liked pop (*n* = 16; 53%), the variable was manipulated to a binary factor, indicating whether the person was a fan of pop or something else.

A model was generated by stepwise logistic regression, performed with the MASS package (Venables and Ripley, 2002).

## Results

One of the subjects had already listened to one of the included albums but did not show outlying values on any variable, thus was included in the analysis.

### Group Level Analysis

Outliers were defined for both the early and late plays. For the early popularity (*M* = 4.02, *SD* = 4.08), one outlier exceeded the mean by three standard deviations (streams = 20.06) and was therefore removed from the sample. This left 23 songs in the early plays sample (*M* = 3.32, *SD* = 2.29). Streams 10 months after release (*M* = 12.94, *SD* = 13.71) also included one outlier exceeding three standard deviations (streams = 59.91), which was another song than the early outlier. Removing this left the late plays sample with 23 songs (*M =* 10.90, *SD* = 9.58).

As the sample contained mostly pop fans (*n* = 16; 53%) and only five R&B fans, the distribution of likeability ratings was examined before continuing the analysis. Remarkably, in subjective ratings the R&B album (*M* = 2.55, *SD =* 0.21) outperformed the pop album (*M* = 2.86, *SD* = 0.21) significantly [*t*(21.43) = −3.67, *p* = 0.001]. This is in line with the both early (*M_Pop_* = 2.18, *SD* = 1.43, *M_R&B_* = 5.58, *SD* = 4.95) and late popularity with the general public, where the two albums differ greatly in favor of the R&B album (*M_Pop_* = 5.77, *SD* = 3.25, *M_R&B_* = 19.0, *SD* = 16.3) as revealed by Wilcoxon rank test (*W_early_* = 23, *p =* 0.004; *W_late_* = 19, *p* = 0.002). Implicit measures between both albums were significantly different on engagement [*t*(19.12) = −2.30, *p* = 0.03] and neural synchrony [*t*(16.40) = −3.79, *p* = 0.002], both showing higher values in the R&B album. Thus, the factor of indicated genre preference was not informative regarding the sample’s true preferences.

#### Correlational Analysis

Normality checks of the dependent variables after the removal of both outliers revealed that both early (*W* = 0.84, *p* = 0.002) and late popularity were non-normal distributed (*W* = 0.70, *p* < 0.001). While early plays did not cross the threshold set by Hair et al. (2010) for skewness (1.19) or kurtosis (3.41), late plays were both heavy-tailed and asymmetrically distributed (*skew* = 2.14, *kurt* = 7.77). Therefore, the later streams had to be log transformed for most analysis to be applicable. The descriptive statistics of all included variables are in Table 1.

**Table 1:** Descriptive statistics of included measures.

Measure	Mean ± SD	Median ± IQR	Normality (*W, p*)
Early Spotify streams	4.02 ± 4.08	2.94 ± 3.12	*W* = 0.67, *p* = <0.001
Late Spotify streams	12.94 ± 13.71	9.34 ± 9.17	*W* = 0.69, *p* < 0.001
Subjective ratings	2.72 ± 0.26	2.72 ± 0.38	*W* = 0.98, *p* = 0.93
Neural synchrony	29.87 ± 0.60	29.74 ± 0.90	*W* = 0.94, *p* = 0.13
Frontal Alpha Asymmetry	0.24 ± 0.60	0.24 ± 0.08	*W* = 0.98, *p* = 0.90
Engagement	0.12 ± 0.07	0.10 ± 0.09	*W* = 0.96, *p* = 0.42

To start with, it was established that the individual subjective ratings were not related to popularity with the general public. Correlations were not significant for both early (*τ* = 0.27, *p* = 0.07) and later Spotify streams (*τ* = 0.36, *p* = 0.02). This indicates that indeed, a simple survey is not sufficiently explaining the popularity with the general population and therefore, a more sophisticated measure must be explored.

For each measure of neural activity, the correlation with popularity was explored. Neural synchrony was not significantly related to early Spotify streams (*τ =* 0.33, *p =* 0.03), but it was significantly correlated to late (*τ* = 0.41, *p* = 0.006) popularity. FAA and engagement were not significantly related to both measures.

In addition to relations to the popularity of music, variables were also compared with each other. Neural synchrony was expected to relate with engagement due to the previous studies but this was non-significant. The early plays, however, were related to later plays (*τ* = 0.51, *p* = 0.0003), indicating that early popularity also may be predictive of later public preference.

#### Linear Model Predicting Popularity

Linear regression was performed on the data where the outliers were removed. The non-normal distributed dependent variables were log transformed only when this was needed for the assumption of normality of residuals.

**Figure 1:**
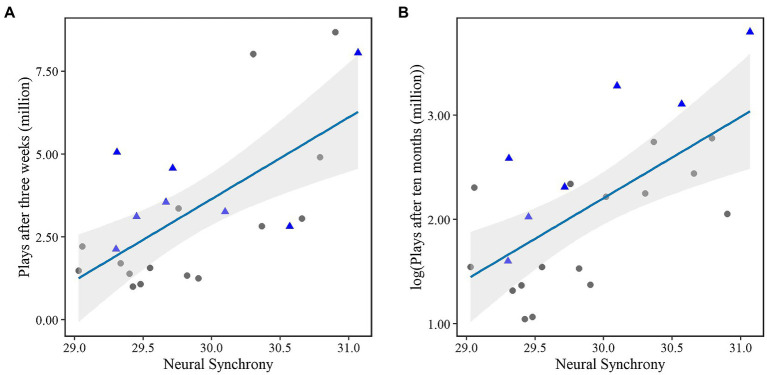
The distribution of singles across neural synchrony and plays **(A)** after either 3 weeks or **(B)** after 10 months log transformed. The blue line indicates the fitted regression line. The gray area indicates the confidence interval. The promotional single releases are indicated by blue triangles.

Following from the correlation analyses, linear regression models were composed with neural synchrony as the main predictor. This confirmed that the proposed neural synchrony could predict 40.4% of the variance ($R_{\mathrm{adj}}^{2}$ = 0.40, *p* = 0.0007) within Spotify plays after 3 weeks. Thus with an 1% increase in synchrony, plays are estimated to increase with 2.4 million (*b* = 2.46, *p* = 0.0007). The distribution is shown in Figure 1A. When the outlier (i.e., the mega-hit) was included, the assumption of normal distributed residuals became violated. The log transformation of early plays was then predicted with 27.93% explained variance ($R_{\mathrm{adj}}^{2}$ = 0.28, *p =* 0.005), where 1% increase in neuro synchrony resulted in 2.08 million more plays (*b* = 0.71, *p* = 0.005).

Even more so, 10 months after the experiment, neural synchrony could still predict 39.3% of the variance ($R_{\mathrm{adj}}^{2}$ = 0.39, *p* = 0.0008). Please note that these streams were log transformed to meet the assumption of constant variance. This indicates that a 1% increase in synchrony can be expected to show an increase of 2.22 million plays (*b* = 0.78, *p* = 0.0008). The distribution is shown in Figure 1B. When the outlier (i.e., the mega-hit on the long term) was included in the analysis, the (still log transformed due to non-normality with residuals) number of streams after 10 months could explain 24.44% of the variance ($R_{\mathrm{adj}}^{2}$ = 0.24, *p =* 0.008), where a 1% increase in neural synchrony would result in 2.13 million more streams (*b* = 0.72, *p* = 0.008).

Including the fact that a song was released as a single, was shown to be very influential in the number of streams over time, as for each album the four most streamed songs were all released as a promotional single. Including the release as a single as factor in the model predicting long-term streams, significantly improved the model (*F =* 13.47, *p* = 0.002) to an explained variance of 61.91% ($R_{\mathrm{adj}}^{2}$ = 0.62, *p* < 0.001), with being released as a single increasing the number of plays with 2.16 million (*b* = 0.75, *p* = 0.002). Models that included the outlier (i.e., the mega-hit) could explain 55.15% of the variance when the factor single was included ($R_{\mathrm{adj}}^{2}$ = 0.55, *p* < 0.001). In the previous model, the weights of synchrony and single were almost equal (*b_synch_* = 0.75, *b_singleyes_* = 0.75), but the model that included the outlier gave more weight to the single release (*b =* 0.96, *p =* 0.0006) as compared to synchrony (*b* = 0.70, *p* = 0.001). For early streams, the fact that a single was released earlier, did not significantly improve the predictability of the model (*F* = 1.86, *p =* 0.19).

To validate the predictive value of EEG measures above and beyond traditional measures, a model was created with stated preferences and the release of a single. For early plays, this model did not meet the assumption of normally distributed residuals (*W* = 0.88, *p* = 0.01) and the comparison therefore could not be made. For late plays, the model of personal liking and the release of a single ($R_{\mathrm{adj}}^{2}$ = 0.33, *p* = 0.007) were significantly improved when neural synchrony was added to the model ($R_{\mathrm{adj}}^{2}$ = 0.62, *p* < 0.001; *F =* 16.09, *p* = 0.0007).

### Individual Level Analysis

On an individual level, the popularity is assessed by the Likert scale ratings that the participants gave after hearing the sample. Since this is an ordinal scale, Kendall correlation was employed for all analyses. The likeability scale was correlated to the two neural measures that could be obtained at an individual level: Frontal Alpha Asymmetry and engagement.

FAA showed a significant correlation with likeability scores (*τ* = 0.10, *p =* 0.0002). Engagement was not significant.

A model that would be able to indicate popularity on an individual level, was constructed by a stepwise logistic regression was performed using the MASS package, where models were selected based on the Akaike information criterion (AIC). This resulted in the following model: 1.83–1.76^*^Engagement + 0.69^*^Artist_R&B_ + 0.46^*^SingleReleased_yes_ (*AIC* = 622.33). Thus, the individual scores increased by a decreased engagement score (*b* = −1.76, *p* = 0.0008). The R&B artist performs better (*b* = 0.69, *p =* 0.001), and songs that were released as a single are also preferred (*b* = 0.46, *p* = 0.04).

## Discussion

Although the predictive capabilities of proposed neural synchrony measure were already validated in numerous contexts and outcome measures, its application in predicting music popularity had yet to be established. As several studies used neural synchrony to predict popularity of movies, and several others measured it on music although not for prediction of popularity, our research perfectly fits the void within the current literature by showing that popularity of music could be predicted by neural synchronization 3 weeks after release and was evenly informative 10 months after release. In addition, the strength of this prediction was valuable above and beyond subjective measures of preference and our results thereby show the importance of employing neural measures to eliminate the biases and preferences that are found in subjective reporting. In the following sections, the results will be discussed more thoroughly and the cognitive processes that might steer the consumers toward music listening will be explained.

### Group Level Analysis

As noted by Boksem and Smidts (2015), it is important that the predictability of neural measures offers insights above and beyond the traditional methods (e.g., stated preferences). Essential aspects thereof include maximizing the generalizability of measurement to real-world situations and always comparing the new measure to the traditional ones. The body of the literature on neural forecasting showed that subjective measures are biased and that neural measures usually outperform stated preferences (e.g., Motoki et al., 2020; Schmälzle et al., 2020; Tong et al., 2020), although some previous studies showed only a miniscule – albeit significant – additional predictive value of neural measurements over subjective ratings (Dmochowski et al., 2014; Boksem and Smidts, 2015).

The distinction between likeability within the sample and the whole population is made by Dmochowski et al. (2014), who reported that neural synchronization was highly predictive of audience-wide ratings, even better than in-sample ratings. Our results indicated the same: Neural synchrony measured within the sample was significantly related to public appreciation on Spotify, however not to subjective likeability scores of the sample itself. The predictive value improved significantly and substantially. This is in line with expectations, as Berns and Moore (2012) also reported that subjective ratings showed no relationship toward the future sales of songs and the previous studies on movie trailers that predicted (box-office) sales of movies (e.g., Barnett and Cerf, 2017; Christoforou et al., 2017). Our results underscore these previous findings and additionally establish that neural similarity is also predictive within the music industry even over several months.

The fact that Spotify streams were predicted 3 weeks and 10 months after their release might seem stunning at first glance. However this robustness also appeared in Christoforou et al. (2017) who could predict the box-office revenues of the premiere based on neural synchronization during the trailer in their sample as well as predicting the actual revenue of the eight weekends following the movie’s release. They explain this long-term effect by stating that neural synchrony might signal memory encoding, which is supported by Barnett and Cerf (2017) who found that higher neural similarity during the trailer was indicative of better recall 10 months later. Thus, it is generally accepted that neural synchrony predicts beyond early popularity indications.

Furthermore, early success is likely to have a disproportionately positive influence on chart positions and thus number of streams (Kaimann et al., 2021). Indeed, in our data, we saw a correlation between early and later streams (*τ* = 0.51, *p* = 0.0002). However, adding the number of early streams to the linear model predicting later streams did not increase its predictive value. Thus, early streams might be strongly indicative of later streams and accordingly show that indeed, it is essential to start off on the right foot when it comes to song releases.

By adding the release of a single as a factor to the model, we incorporated the effects that the promotion of these songs might have had on the number of plays. Studies have been dealing with these external factors differently; most of them do not even mention the fact, whereas Christoforou et al. (2017) divided the box-office revenues by the total budget of the movie in order to account for the variability of marketing capacity and reach. The fact that single release accounted for such an increase in predictability resolves most likely from the fact that the release of a single is associated with a longer time in the charts of digital streaming platforms, which has a powerful sway on the number of streams (Kaimann et al., 2021).

Our data indicates that the combination of higher neural synchronization on the track as well as the release as a single constituted an increased number of plays. Therefore, we argue that a song has to have the “mega-hit potential” and then, single release might be able to increase its popularity. This proneness to become a hit could potentially be estimated by neural similarity measures. As several studies have already indicated that neural synchronization increased with specific aspects of music, such as rhythm, melody, tempo, and phrase repetitions (Czepiel et al., 2020; Kaneshiro et al., 2020), it is agreed that neural similarity identifies emotions at the level of musical features and we hypothesize it will therefore serve as a strong predictor of mega-hit potential.

Additionally, to the effects shown here, the timing of the study adds real value to the results. Most studies on film trailers tested movies that were already released. A little late to the party, as there might have been other factors impacting the consumers’ implicit reaction to movie trailers, such as social buzz or promotions. Our study has started on the first day of the album releases, in order to exclude these contributing factors as much as possible. This is also shown by the fact that only one respondent had heard one of the albums already. Therefore, the results could not be influenced by any prior knowledge of either respondents or experimenters and represent therefore a true measure of hit potential.

Several studies have indicated that neural synchrony is linked to collective engagement (Dmochowski et al., 2012; Chan et al., 2019). In the present data, no relationship between intra-subject engagement and inter-subject neural synchrony was found. This could be explained by differentiating definitions and measures of engagement. For example, Dmochowski et al. (2012) manipulated engagement by showing the same movie clip once in regular order and once scrambled, thereby deteriorating the narrative. Although this is expected to decrease engagement, it is not based on the same definition of engagement incorporated by Berka et al. (2007) and thus, our definition of engagement might be built on other grounds. The use of different neural measures and methods may also contribute to the inconsistent findings. More research is needed to establish the precise relationship between ISC and engagement.

Additionally, engagement and FAA both did not contribute to the prediction of population-wide popularity. Such a relationship was expected for the FAA, as it has predictive value over subjective ratings when it comes to sales (Baldo et al., 2015); however, the hypothesis for individual engagement in this area was more agnostic as there exist no prior studies demonstrating that task engagement predicts popularity. Future research might establish the exact relation between these measures and larger audience popularity.

### Individual Level Results

On a group level, other brain measures were not significant to predict general popularity. However, on a more individual level, we found that FAA was related to the subjective likeability ratings and in addition, engagement was an important predictor when combined with the factors artist and single release. Here, we propose why these measures may explain subjective ratings but not the larger audience popularity.

FAA has been established as an indicator of approach-avoidance motivation, increasing with higher stated likeability (Davidson et al., 1990). The metric has been extended to several fields of applied research, including neuromarketing, diagnostics, brain computer interfaces, and therapeutic tools (Briesemeister et al., 2013). Vecchiato et al. (2011) showed that FAA was related to self-reported pleasantness of commercial videoclips, and larger audience popularity was assessed by Shestyuk et al. (2019) showing that FAA during a TV show episode correlated to its viewership and Twitter volume. Especially, Baldo et al. (2015) showed the superiority of FAA over subjective ratings when correlating to the sales of a shoe model. It was therefore hypothesized that FAA would relate to subjective ratings, but also would generalize outside the sample. While we confirmed the first hypothesis, the latter one remains uncertain.

For the engagement measure, we found the same trend: It was not related to larger audience preferences but instead was included in the model predicting stated preferences. This might be explained because the calculation of the engagement metric is done on an individual level – as is the case with FAA. Engagement values are derived from a model that returns probability of high engagement (Berka et al., 2007), specifically tailored to the brain responses of that person due to the benchmarking tests that were done beforehand. This tailoring might decrease the generalizability across subjects thereby making engagement a measure best suited for individual preferences. This hypothesis, however, warrants further investigation. A fruitful avenue for further research in this regard would be to actually manipulate the levels of engagement in order to deepen our understanding of the relationship between individual and group level preferences at various levels of engagement.

### Limitations

External factors, such as promotional budget and whether the song has been in charts or shared playlists, have not been included in the study, which might provide a true limitation. Social proof is an important factor in music preference (Berns et al., 2010) as charts are highly valued by consumers (Kaimann et al., 2021); tracks listed in the charts benefit from increased visibility and perceived quality. Moreover, the social sharing component of Spotify and the customizable playlists have further enabled customers to cherry-pick the best outputs from a wide range of music (Kaimann et al., 2021), which may exponentialize popularity on Spotify. In addition, the difference between album sales and Spotify streams is that streams are affected by repeat consumption, while album sales are not (Kaimann et al., 2021). Therefore, these external factors might have an excessive impact on the number of streams and it is extremely difficult to detangle their relations. A future study where these measurements are included might provide better insight in the true drivers of streaming popularity and extend the understanding of its relationship to neural synchrony in doing so.

The prediction of popularity with the larger audience has severe implications for the music industry, enabling them to select which song should be released as a single, which has to get more promotional budgets and whether they will invest in an artist at all. However, we should be cautious in extrapolating the current study’s conclusion to the entire music industry, as the downside of our research is the inclusion of only two albums. From an industry’s perspective, it would be invaluable to be able to predict the mega-hit from one album, as in real-life situations where the producing artist exchanges ideas with the record label on which songs carry the most hit potential, thus warranting the largest slice out of how the marketing budget is spent across the tracks. To further explore this situation, we evaluated the predictive power of neural synchrony when only one album was included in the dataset. While adding the artist as a factor in the model was not significantly increasing explanatory power, t-tests showed that the number of streams was significantly different between the two albums. The neural synchrony, however, was not significantly different between the albums. Linear models constructed for the prediction of the R&B albums early plays were significant (*R*_adj_ = 35.99, *p* = 0.02) where neural synchrony positively impacts the number of streams 3 weeks after album release. However, for the late plays, separate models for both artists were insignificant. Thus, selecting the mega-hit including only one album in the analysis might be less predictive, most likely because of the small dataset that comes with including only one album.

Building a broader database of various songs and their neural synchrony and popularity might therefore diversify the data and thereby increase robustness of predictive power from the data. Since the present study did not test on a whole diversity of artists as was the case with Berns and Moore (2012), the predictive power of EEG-based neural synchrony across multiple artists and diverse genres has still to be established.

Additionally, the distribution of genre preference in our sample might be another limitation, as most participants rated pop (*n* = 16; 53%) as their favorite genre, and the sample contained only 5 R&B fans (hip hop = 8, rock = 1). As our results showed that the R&B album scored higher on both subjective ratings and neural measures, it was deduced that most likely the participants did not correctly state their preferred genre. Another explanation might be the relative unpopularity of the pop album as its number of streams were also significantly lower. Either way, the distribution of genre preferences within the sample and the genres of the tested albums might have impacted the outcomes. Alluri et al. (2013) showed that ISC varies to different genres of music, related to other brain regions, which provides the urge to explore this aspect even more.

Other limitations with regard to convenience sampling are represented by the unequal gender distribution (7 male and 24 female respondents). Neural synchrony calculations over different groupings, such as gender, can infer different conclusion: Barnett and Cerf (2015) showed that some content in a commercial produced similar responses for both genders, whereas other content had dissimilar effects. Gender effects are also found with music listening as the Spotify algorithm classifies on gender (Werner, 2020). Although the aggregate neural synchrony measure might be modulated by a skewed gender distribution, the relationship with Spotify plays was still established with the sample and this proves generalizability of this measure. For future researches, the sample should be balanced in order to rule out the possible confounding effects of gender distribution.

### Location and Frequency of Neural Synchrony

Spatial localization is not the main strength of EEG. Several studies have been conducted in order to establish a more precise definition of locations of neural synchronization during naturalistic stimuli, but so far the results have yet to yield consensus among researchers. Where Barnett and Cerf (2017) and the present research report ISC over alpha frequencies in the central electrodes, beta, and gamma frequencies are also mentioned in Dmochowski et al. (2012) and Christoforou et al. (2017) relating to Boksem and Smidts (2015). The underlying motivations and therefore explanations behind neural synchrony might depend strongly on the included frequencies and localization.

Since we hypothesized the central electrodes within the alpha frequencies, we are basically evaluating the activity patterns of the respondents’ mu rhythm, which most likely originates from the somatosensory cortex related to movement and movement preparation (Pfurtscheller and Da Silva, 1999). This sensorimotor synchronization in the alpha band is also called mu suppression, and several studies showed that music listening was related to this activity within motor areas (Chen et al., 2008; Grahn and Rowe, 2009; Ross et al., 2016). The coupling of music listening to motor areas makes sense as the presence of a beat usually causes spontaneous synchronized movements like toe tapping or head nodding (Janata et al., 2012) and beside its prevalence during action performance, mu suppression is also found during observing a movement, and therefore, it might be linked to the mirror neuron system. Although this link still needs robust evidence (Hobson and Bishop, 2017), it might explain the coupling of hearing a beat and synchronizing movement to it – either imaginary or real dancing. And these presumably will be similar across listeners.

Besides these sensorimotor coupling, alpha frequencies are also linked to attention and memory tasks (Klimesch, 2012). Johndro et al. (2019) showed how rhythm increased attention for targets that are presented on-beat as compared to off-beat and this may strengthen memory encoding. Since several studies showed a relation between neural synchrony across participants and attention, this might also provide an explanation for neural similarity results on musical data. The real underlying activity might be further explored.

Previous research predicting music popularity was conducted with fMRI, which has higher spatial resolution compared to EEG. Berns and Moore (2012) found significant results for activation in the ventral striatum/nucleus accumbens (NAcc) during listening to the music samples. The NAcc is anatomically connected to the orbitofrontal cortex (OFC), which was related to purchase decisions as found in the previous studies (Knutson et al., 2007). Especially, listening to non-hits resulted in low-OFC and low-NAcc activations. For personal preference, the linkage was also significant. This connection is usually explained as reward anticipation and correlated to the EEG measure of FAA according to Gorka et al. (2015). However, FAA was not a significant predictor of population-wide popularity in the current study.

Another fMRI study predicting popularity, but for movies, compared the predictability of EEG and fMRI. Dmochowski et al. (2014) found that brain regions in the lateral temporal cortices were highly correlated with EEG neural reliability and an area of parietal cortex including the superior parietal lobule and precuneus. Meanwhile, a significant negative covariation between neural reliability and BOLD activation was found in a region of medial prefrontal cortex that includes the anterior cingulate cortex, as well as the left inferior frontal gyrus.

Other EEG studies (e.g., Boksem and Smidts, 2015; Christoforou et al., 2017) selected electrodes based on the most discriminating spatial filters in the beta and gamma bands. Beta bands showed a mid-frontal cluster focused on AFz, F2, FC1, FCz, and gamma band showed a fronto-central cluster surrounding frontal, fronto-central, and central electrodes (Boksem and Smidts, 2015). These provide a reason for future research to also explore the relation of the (pre-)frontal cortex within EEG-based neural synchrony when listening to music.

In addition to the EEG’s reduced spatial resolution compared to fMRI, the current study was conducted with a nine-electrode EEG system, without channels at fronto-central locations, which therefore could not be include in the analysis. However, the validation that this comfortable, relatively affordable and easy-to-apply nine-channel EEG still provides predictive value for popularity, is very important for the end-users in the neuromarketing field. The commercial use is motivated by comfort both for the researcher and respondent, and our results are therefore very valuable for this community.

### Future Research

The previous paragraphs most logically deduce the need for a follow-up research where more diversity in musical genres and artists is included. Authors suggest constructing a database wherein the predictive power of neural synchrony over subjective ratings might be further explored and the relationship between them validated on a broader musical library. As noted under the previous limitations, it is important to include as many external variables as possible; such as liking and sharing rates, time in charts, and appearance in public playlists since these have substantial effect on the number of digital streams (Kaimann et al., 2021).

Additionally, the selection of the target audience might be evaluated. The current study engaged in convenience sampling, but it might be that selection on musical taste is preferred. Since neural similarity is a group-level metric, there might be some respondents disliking a certain genre and consequently downsizing group level neural synchrony. Of course, this can be prevented by checking the pairwise correlation before aggregating them. However, including only fans in the data would in theory lead to a better representation of the target audience. This might be especially valuable in the use case we presented earlier, where record labels want to determine the division of marketing budget across songs on the album – particularly for niche market music. The true increase in predictive value by recruiting the album’s corresponding target audience has yet to be established.

Furthermore, Schmälzle et al. (2020) noted that multiple studies have compared neural synchrony values; however, the dynamic fluctuations within a stimulus are rarely understood. Moment-to-moment neural responses might vary across audiences (Barnett and Cerf, 2015), and Dmochowski et al. (2012) also proposed a more integrated look at the peaks within neural synchronization to explain their origin from the naturalistic stimuli. Evaluating moment-to-moment ISC might reveal the underlying processes within the music, such as its relationship to beat, lyrics, or emotional state.

Elaborating on this suggestion, a future study could extend our findings by implementing valence and arousal measures to the data, in order to predict emotional experience during music listening. Especially, since listening to music is usually an emotional experience. Further research on emotions, perspective taking and neural ISC is provided by Jääskeläinen et al. (2020a,b). Classification of emotions across participants can be improved by adding inter-subject correlations to the feature space as shown by Chan et al. (2020) and Haxby et al. (2011) and thus may be an interesting elaboration of the current research.

Additionally, Dmochowski et al. (2012) made the distinction where neural synchrony is defined as an indication of agreement within a group of individuals, conveying the fact that multiple viewers must be experiencing the stimulus similarly, as these are modulated by the content of the stimulus inter-subject correlation (ISC). On the other hand, they defined intra-subject correlation which indicated how reliably a scene elicits a response within the same subject. The neural response varies within and across individuals due to their subjective evaluations and uniqueness of brain structure with each individual, and therefore, it may be interesting to compare our between-participant results also within the participants in order to evaluate their responses to repeated presentation of a musical sample and in addition to establish a more integrated view of the matter. Functional Connectivity measures are widely implemented in music in order to evaluate emotions (e.g., Daly et al., 2014; Naser and Saha, 2021) and would provide a valuable reference point to compare between and within neural similarities.

### Practical Implications

As the research is directly related to a real-world problem, the practical implications deserve a proper discussion. While we do encourage the use of neuromarketing tools for prediction of popularity, authors note that subjective ratings might still provide valuable insights and should be considered next to implicit ones.

Since digital streaming now accounts for 85% of music industry revenue in the United States (Recording Industry Association of America, 2020), the contribution of the present study is substantial and of great importance to the music industry. Our results show that the combination of high neural similarity and the release of a single predicts the popularity on Spotify greatly. While it was known that single release significantly increases its digital streaming (Kaimann et al., 2021), the effect thereof may be even improved by choosing the right song to turn into a single. Since the functions and algorithms of digital streaming platforms may easily exponentialize the number of streams as soon as the song is picked up by a chart, early popularity is almost essential for later popularity (Kaimann et al., 2021). This implies that the choice of single has to be right from the beginning, stressing the importance of an accurate prediction. Neural synchrony provides the perfect opportunity to pinpoint the hit potential among a musical database and thereby might assist the music industry in dividing its marketing budgets. Before the release of an album, the record label can value the songs based on their neural synchrony value and in doing so divide the marketing budget properly and choose the highest scoring songs to release as promotional singles.

The findings can also be extended beyond music as neural synchrony is already an established predictor for movie liking (Chan et al., 2019) and revenues (Barnett and Cerf, 2017; Christoforou et al., 2017). Besides entertainment, Dmochowski et al. (2014) and Chan et al. (2019) showed that advertising industries might also benefit from neural synchrony as a neuromarketing metric. In advertising, the success of a commercial is largely dependent on either brand image (i.e., brand recall) or motivation to buy. Besides commercial use, health messages, for example, regarding excessive alcohol use can also benefit from predicting their impact with neural synchrony (Imhof et al., 2020). Thus, the application of this metric in advertising would be valuable.

Last but not least, our results present itself in the middle of the void that existed in the literature where both fMRI and EEG ISCs were related to popularity, but music was only evaluated by fMRI. That flagship study of Berns and Moore (2012) has been an example in neuromarketing for years, and our validation of the predictive approach using EEG provides great theoretical contributions. Additionally, the timing of the experiment within the first days after the album release is a unique aspect that is not shown in the previous research on film trailers. We therefore provided the results that bridge the gap in this line of research and provide a great theoretical contribution to the field of neuroforecasting, EEG-based neural similarity, and musical popularity prediction.

## Conclusion

Thus, our results provide strong evidence for neural synchrony to be applicable as a predictor of population-wide digital streaming numbers and correspondingly the popularity of music with the large audience. Additionally, individual preferences could be explained with Frontal Alpha Asymmetry or engagement. The practical impact lies with the music industry in the first place, enabling them to accurately assess the hit potential from their music samples and thereby distributing their resources more efficiently. Additionally, this metric might be applicable to neuromarketing research as forecasting consumer preferences lie at the heart of this discipline. By showing the predictive value of EEG-based neural synchrony on music, this research fills the void in the existing literature where previously popularity prediction had been done with movies, and music predicting had been done only with fMRI. The use of EEG and the application on music thus make this research unique within the existing literature.

## Data Availability Statement

The raw data supporting the conclusions of this article will be made available by the authors, without undue reservation.

## Ethics Statement

Ethical review and approval was not required for the study on human participants in accordance with the local legislation and institutional requirements. Prior to the experiment, participants singed an informed consent in accordance with NMSBA Code of Ethics and EU GDPR guidelines.

## Author Contributions

TB, NF, and DP contributed to the conception and design of the study. NF and DP led the data collection process. NL performed the statistical analysis and wrote the first draft of the manuscript. All authors contributed to the manuscript revision, read, and approved the submitted version.

## Conflict of Interest

TB, NF, and NL were employed by the company Unravel Research. Authors’ employment do not depend on the outcomes or publication of this study. The authors declare no financial or otherwise competing conflicts of interest.

The remaining author declares that the research was conducted in the absence of any commercial or financial relationships that could be construed as a potential conflict of interest.

## Publisher’s Note

All claims expressed in this article are solely those of the authors and do not necessarily represent those of their affiliated organizations, or those of the publisher, the editors and the reviewers. Any product that may be evaluated in this article, or claim that may be made by its manufacturer, is not guaranteed or endorsed by the publisher.
